# The intersubjective endeavor of psychopathology research: methodological reflections on a second-person perspective approach

**DOI:** 10.3389/fpsyg.2014.01150

**Published:** 2014-10-17

**Authors:** Laura Galbusera, Lisa Fellin

**Affiliations:** ^1^Clinic for General Psychiatry, University of Heidelberg, Heidelberg, Germany; ^2^Division of Psychology, University of Northampton, Northampton, UK

**Keywords:** intersubjectivity, social understanding, psychopathology research, methodology, second-person perspective

## Abstract

Research in psychopathology may be considered as an intersubjective endeavor mainly concerned with understanding other minds. Thus, the way we conceive of social understanding influences how we do research in psychology in the first place. In this paper, we focus on psychopathology research as a paradigmatic case for this methodological issue, since the relation between the researcher and the object of study is characterized by a major component of “otherness.” We critically review different methodologies in psychopathology research, highlighting their relation to different social cognition theories (the third-, first-, and second-person approaches). Hence we outline the methodological implications arising from each theoretical stance. Firstly, we critically discuss the dominant paradigm in psychopathology research, based on the *Diagnostic and Statistical Manual of Mental Disorders* (American Psychiatric Association, 2013) and on quantitative methodology, as an example of a third-person methodology. Secondly, we contrast this mainstream view with phenomenological psychopathology which—by rejecting the reductionist view exclusively focused on behavioral symptoms—takes consciousness as its main object of study: it therefore attempts to grasp patients’ first-person experience. But how can we speak about a first-person perspective in psychopathology if the problem at stake is the experience of the other? How is it possible to understand the experience from “within,” if the person who is having this experience is another? By addressing these issues, we critically explore the feasibility and usefulness of a second-person methodology in psychopathology research. Notwithstanding the importance of methodological pluralism, we argue that a second-person perspective should inform the epistemology and methods of research in psychopathology, as it recognizes the fundamental circular and intersubjective construction of knowledge.

## INTRODUCTION

Psychology, as a discipline, is mainly concerned with knowing others’ *minds*^1^. The problem of social cognition is therefore crucial to any psychological research enterprise, and the way we conceive social understanding influences the way we do research in psychology (Reddy, 2008). Questions regarding the possibility of understanding other persons, the way social understanding works and the influencing factors that play a role in this process are tightly related to epistemological and methodological issues such as: the validity of our claims in doing psychology research; the development of a proper methodology to understand our object of study; and the way we should frame and interpret our results according to the context in which they arise. These questions take a particular turn in the field of psychopathology research, where we do not only deal with other minds, but with minds especially experienced and constructed as *different*^2^, because of their distress and not-ordinary experience.

Psychopathology is a controversial field of research characterized by often very polarized views. Mainstream studies focus on “mental disorders,” considered as inherent to individual “minds” (brains) suffering from psychological distress or impairment (or even biochemical or genetic deficits). At the opposite edge of this continuum, other approaches try to describe and understand the contextualized and embodied meaning of the distress (as for instance, phenomenological psychopathology) and look for its socio-relational and interpersonal features, origins, and functions^3^; or the most radical positions (as for instance, anti/critical psychiatry and many systemic theorists) may even deny the existence of such a phenomenon as individual psychopathology in favor of a more social and relational understanding of distress^4^. Either way, psychopathology research has to deal with making sense of the experience of otherness, difference, and alterity, even when the aim is to deconstruct what is considered to be a labeling process. Before defining, classifying or constructing etiological theories, psychopathology research therefore needs to deal with the primary task of understanding others, but even more *different* others. The core methodological issue at stake here is therefore: how can we understand others, in their difference? To explore this question, it seems logical to bring together theories of social understanding with approaches to psychopathology research and this is what we do in the present paper.

Since decades the problem of social understanding has been at the core of the contemporary debate in the cognitive sciences. Different theories and frameworks have been proposed to account for this phenomenon and still the debate remains controversial (see, for instance, Gallagher, 2012; Dullstein, 2012). These theories look at how we understand others from three different perspectives: a third-person perspective (Theory-Theory), a first-person perspective (Simulation-Theory), and a second-person perspective (e.g., Interaction Theory, IT). We critically review these perspectives on social cognition, highlighting and discussing their core claims. Though, it is important to stress that our aim is not to offer an additional contribution to the debate, but rather to take this very debate as a starting point for some methodological reflections relevant for psychology and the cognate disciplines. In particular, we look at the kinds of encounters that take place in the context of psychopathology research as a paradigmatic case of the methodological issue at stake; because here, as above mentioned, the relation between the researcher and the object of study is characterized by a major component of “otherness.” Then, we highlight a number of methodological implications for psychopathology research that necessarily arise from our critical discussion of approaches to social cognition.

Although we generally acknowledge the need for methodological pluralism and we do not see these perspectives as mutually exclusive, we adopt a rather critical stance: in what follows, we will outline some shortcomings of the third- and first-person perspectives. If these shortcomings hold for a general theory of social understanding, then they should be relevant for those methodological issues in psychopathology research as well. Dixon (in Stanghellini, 2007) identified a dilemma inherent in the science of the mind, “Is the science of the mind in fact to be a science of the mind, or a science of something else, such as the brain or behaviour? Is it to be ‘science by analogy’ or ‘physical science proper’?” (p. 69). The same question may be posed for a “science of suffering minds/souls” (from the Greek etymology of the word “psychopathology”). In exploring these questions, we will therefore point to a second person alternative as a promising (although not unproblematic) methodology of psychopathology research. Following Stanghellini’s (2007) answer to Dixon’s question:

The phenomenological perspective, and specially the second-person mode, advocates that the context of the clinical encounter^5^ should be one of co-presence (and not of dominance) with the aim of understanding (and not labelling), i.e. negotiating intersubjective constructs, and looking for meaningfulness through the bridging of two different horizons of meanings. (p. 70)

## SOCIAL UNDERSTANDING AND METHODOLOGY IN PSYCHOPATHOLOGY: FROM A THIRD-PERSON PERSPECTIVE TO A FIRST-PERSON PERSPECTIVE

### THE THIRD-PERSON APPROACH OF MAINSTREAM PSYCHOPATHOLOGY RESEARCH

Research in psychopathology mainly focuses on understanding the causes, correlates, and consequences of psychological disorders. It is commonly based on the *Diagnostic and Statistical Manual of Mental Disorders* (DSM; American Psychiatric Association, 2013) and its diagnostic categories, consisting mainly in lists of symptoms. Despite its claim of a-theoreticity (since its third edition), the DSM diagnostic system—and therefore mainstream research in psychopathology—relies on an epistemology of logical empiricism and on physicalist ontology (Schwartz and Wiggins, 1986; Parnas and Bovet, 1995; Parnas et al., 2013; Parnas and Gallagher, in press). Symptoms and mental states are reified and seen as ontologically independent atomic entities, as material thing-like objects. Psychological reality is constructed “out there,” independent of any human perspective, as if it could be known objectively through empirical observation (e.g., medical test) and logical thinking by an “external objective expert” (Parnas and Bovet, 1995; Parnas et al., 2013). By a deceptive mistake that Husserl (1970) would call “naive objectivism” of the life sciences [or, a “game of semantics” in Timimi (2013) words], symptoms become much more than descriptive constructions: they are reified providing the illusion that the disorder itself exists as a natural object. This easily leads to etiological theories that link mental distress to supposed biochemical or genetic causes (and therefore, mostly pharmaceutical interventions). In this approach to diagnosis, individuals are equated to their diagnostic label and therefore stigmatized or even alienated and dehumanized. To borrow Simblett’s (2013) words, it is possible “to understand DSM as a textual codification of power/knowledge that creates a version of reality, individuality and what is known about the nature of mental illness. But only one possible version” (p. 116). Despite attempts to unpack and deconstruct this discourse, revisions to DSM are concerned almost exclusively with its criteria and thresholds. This also shapes and limits the field of possible research in psychopathology: only approaches constrained within the boundaries of mainstream research are viable, therefore reinforcing its power and the knowledge imbalance in the research encounter (Irarrázaval and Sharim, 2014).

With some notable exceptions of systemic, psychodynamic, constructivist, and phenomenological authors, psychopathology research is mainly based on quantitative methods: symptoms, mental states, performances, personality traits, or neurological features (etc.) are operationalized as measurable variables to be statistically correlated with specific diagnoses (Sher and Trull, 1996). The source of diagnostic data are mainly structured interviews (or even self-reports) which limit the person’s freedom of expression by severely restricting their possible responses. They are based on the same epistemology: de-contextualizing and fragmenting the other’s experience into a list of internal mental states and external behaviors that may be counted as present or absent or rated by their intensity/severity. This in order to fit these behaviors and mental states into the rigid and pre-packed diagnostic classification, which is then as well treated as a variable for research purposes. The attention is mainly on the verbal and cognitive level, the experience is dis-embodied and de-contextualized rather than socially situated (Cromby, 2012).

If we look at this research paradigm from the view of cognitive science we may notice many parallels with the Theory of Mind theory (TT) of social understanding also referred to as a third-person approach. The TT is based on the following main assumptions: others’ mental states^6^ are hidden, we do not have direct access to them (mind–mind gap or “inner world hypothesis”), and we therefore need some extra cognitive processes in order to infer the mental state of the other (mentalizing supposition)^7^. In inferring and theorizing about other minds we need to refer to common sense, i.e., folk psychological theories about how mental states (beliefs, desires, intentions) inform the behaviors of others (Malle, 2004). Observation becomes the evidence for theorizing and this constitutes our everyday stance toward others (spectatorial supposition): we always observe others’ behavior with some degree of detachment, trying to infer their mental states from a third personal stance (Gallagher, 2001).

If we now look back at the mainstream methodology in psychopathology research we may notice similar assumptions at the basis of this paradigm. Variables such as symptoms, behaviors, performances are considered as an objective reality that can be observed by a detached researcher (expert); the mental states of other persons are often inferred from behavioral cues or even neurological features according to already existing theories. Therefore, this kind of approach may commit several errors and take recourse to biases, as widely illustrated by decades of attributional research.

The experience of the other person is usually directly accessed (or “assessed”) by the expert position through structured interviews, where the researcher is considered as detached from the patient and his task is to infer the patient’s state of mind, which is assumed as objective and a-contextual. A paradox seems to be entailed in this approach, as Reddy (2008) pointed out in her criticism of TT: if, on one hand, we adopt an empiricist view—where the only way to know things is through experience given to our senses—and, on the other hand, we claim that other minds are not accessible to our experience but rather hidden behind behaviors (mind–mind gap), knowing other minds results in a logical impossibility. Similarly, how can we claim to adopt an empiricist methodology if the object of study (patient’s mental states) needs to be inferred?

### REDISCOVERING SUBJECTIVITY AND PATIENTS’ FIRST PERSON PERSPECTIVE

Parnas et al. (2013) strongly criticize the third-person approach in psychopathology research starting from a critical discussion of its subject matter: “The object of psychopathology is the ‘conscious^8^ psychic event’, and psychopathology involves and requires an in-depth study of experience and subjectivity” (p. 271). They stress the importance of focusing on first-person experience and subjectivity in the study of psychopathology, without denying the usefulness and necessity of a methodological pluralism. In fact, although useful for understanding mental phenomena, the study of neural substrates, behavioral descriptions and task performances always assumes its relevance in relation to the conscious level: the researcher’s interest is never in brain events and behaviors *per se* but in their relationship with mental phenomena and experience (Nordgaard et al., 2012). The call for a first-person approach (Parnas et al., 2013) therefore not only points to the fact that first-person experience should be the object of study for psychopathology research—ultimately, the third-person approach is an attempt to understand others’ experience as well, even if in a reductionist and fragmented way—the focus is rather on the nature of this object and on how it can be understood. The issue at stake is thus primarily ontological and epistemological.

Embracing a phenomenological view, consciousness cannot be ontologically considered as atomistic in nature because it is an ever-changing flow of mutually interdependent phenomena. Consciousness is a Gestalt, a meaningful whole that cannot be reduced to an aggregate of parts, a sum of “mental objects.” Symptoms and behaviors are not meaningless entities from which we can infer hypotheses about mentality and create a-contextual definitions and quantifications; they always have a meaning^9^ that derives from the total state of consciousness, embodied and embedded in the environment. Therefore, as maintained by Parnas et al. (2013):

It is crucial to understand phenomenal consciousness (subjectivity) as the overall field, ground, or horizon within which all “manifestation” or “presencing” of the objects of our awareness occurs. Consciousness, the phenomenal manifestation of thoughts, feelings, and perceptions, is not some kind of complex spatial, 3-dimensional object but a lived reality, a presence to itself and the world: “psyche,” writes Jaspers, is “not […] an object (…) but ‘being in one’s own world,’ the integrating of an inner and outer world.” Consciousness manifests itself as a “becoming,” a temporal “streaming” of a unity of intertwined experiences. (p. 274)

The phenomenological method introduced in psychiatry by Jaspers and other influential psychiatrists, such as Binswanger, Minkowski, and Blankenburg (and then expanded toward a more interpersonal perspective by Laing), represented the primary instrument for investigating and describing the first-person experience of patients (Bürgy, 2008). It is therefore often referred to as a first-person approach, mainly because of the clear shift in the consideration of the psychiatric object: the focus is on consciousness as a whole, grasped through an in-depth exploration of patient’s first-person experience (Fuchs, 2010). As Parnas et al. (2013) maintained, notions such as self, ownership, reality, rationality, etc. are of core importance for psychopathology research, therefore rendering it necessary to focus on subjectivity and the first-person perspective.

Yet, epistemologically, one may wonder what a first-person approach might mean in a context where the object of study is actually the experience of the other. As Parnas et al. (2013) recognized: “(…) A second domain concerns how and to what extent is a psychiatrist able to access the patient’s mind and reconstruct his experience” (p. 274). When shifting from the issue of the object to that of the method, can we still speak of a first-person perspective? What does it mean then, to have a first person understanding of other minds? And how should we therefore conceive a first-person methodology? In what follows, we will try to clarify this issue and shed some light on terminology by referring back to the social cognition debate.

### A FIRST PERSON METHODOLOGY FOR UNDERSTANDING OTHERS

The first-person perspective on social understanding in cognitive science has been defended by the Simulation Theory (ST). Although sharing the same basic assumptions of TT, ST differs from the latter in the way the gap between two minds is filled. As for TT, others’ mental states are considered as hidden: we lack direct access to them and our everyday stance toward the other is still an observational one. The problem is therefore still framed in the same way: how, when observing others, can we figure out their hidden mental states?

Instead of inferring mental states on the basis of folk psychological theories, ST claims that we need to simulate within ourselves the mental state of the other, as if we were them, in order to understand it.

In philosophy, this process has also been called the argument from analogy: by analogy with our own experience, we infer that other bodies must also experience the same sort of mental states that we have.

Although some (e.g., Goldman, 2005) conceive of this process of simulation as a mentalizing one, other approaches (Implicit Simulation Theories) maintain that we implicitly attune with others at much more basic levels. For instance, drawing on the neurological basis of mirror neurons, some ST proponents claim that through the implicit recognition of similarity between our actions, we are immediately able to reproduce the mental state of the other person when we see the action they perform (Gallese and Goldman, 1998).

Within the social cognition debate, ST has already been widely criticized under many aspects. Gallagher (2012), for instance, pointed out the contradiction in putting the very notion of simulation at the basis of social understanding:

One can see the starting problem clearly, for example, in Goldman’s description of the first step involved in running a simulation routine. “First, the attributor creates in herself pretend states intended to match those of the target. In other words, the attributor attempts to put herself in the target’s ‘mental shoes”’ (Goldman, 2005, p. 80). This first step seems tricky. How do I know which pretend state (belief or desire) matches what the other person has in mind. Indeed, isn’t this what simulation is supposed to deliver? If I already know what state matches the target, then the problem, as defined by ST, is already solved. (p. 207)

As we will mention later on, while describing Gallagher’s own theoretical proposal for social understanding, what he finds missing in first-person accounts is the recognition of contextual knowledge and interactive processes as necessary and constitutive parts of social understanding. Reddy (2008) further argued that a ST of understanding does not even solve the problem of the gap between two minds, as it basically relies on an overgeneralization of one case (one’s own experience). Although in ST the focus is more on experiencing than on theorizing, the experience on which I base my knowledge of the other can only be my own: it is still an attribution based upon the self (Reddy, 2008).

The argument from analogy for explaining social understanding is considered controversial in the phenomenological literature; as we will contend in the next section, since Husserl’s understanding of empathy as the primary mode of social understanding, it is clear that phenomenological theories are rather coherent with a second personal mode. Although a simulationist understanding of empathy, as an “as if” awareness of the other person, has been repeatedly rejected in phenomenological theories, it can still in some cases inform the methodology of phenomenological psychiatry, which is nowadays sometimes referred to as first personal in this sense (Stanghellini, 2007, 2010; Fuchs, 2010).

For instance, in Jaspers’ (1997) *General Psychopathology*^10^ the process of understanding the patient has often been described as an “imaginative actualizing” of the other’s experience (Fuchs, 2010; Wiggins and Schwartz, 2013): in order to understand others, we need to relive (*nachleben*) in ourselves their experiences. Starting from the assumption that the best evidence of mental life is self-reflection, the best way to access what cannot be immediately present to us (others’ experience) is to make it present through a process of imaginative identification (Wiggins and Schwartz, 1997, 2013). Therefore, by intuitively representing the other’s psychic states, we can grasp what it is like to be like him/her: a transpositional movement that actually follows the structure of analogy (Stanghellini, 2007). This process of empathically putting oneself in the other’s place in order to understand him/her, presupposes a “bracketing” of one’s own assumptions and prejudices, in order to get as close as possible to the original experience of the other. Although we acknowledge the importance of this methodological step, the epistemological concern related to a first-person methodology (as for the criticism of a ST of social cognition) is whether I am projecting my own experiences onto the other, which may go with the risk of transforming understanding into mere speculation (Stanghellini, 2007; Wiggins and Schwartz, 2013), or determining, rather than understanding, the other (Reddy, 2008).

This leads us to the exploration of what has been proposed as an alternative in cognitive science: a second-person perspective. Before entering into the core of the methodological discussion on this regard, it is worth looking at how, in the cognitive sciences, this approach has been defined and constructed through different contributions. We will do this in the following section in order to move, in Section “Methodological Implications for a Second-Person Psychopathology,” to the methodological discussion, where we draw some methodological implications for psychopathology research directly from each main claim of the second-person approach in cognitive science.

## A SECOND-PERSON APPROACH TO UNDERSTANDING OTHERS

The second-person approach offers an alternative explanation of social cognition based on a firm refusal of the body–mind gap and the mind–mind gap. It is often referred to as Interaction Theory (Gallagher, 2001), which draws on a phenomenological understanding of social cognition. Nevertheless, different authors contributed to defining this perspective, rendering it more elaborate and complex.

Phenomenological approaches challenge the basic assumptions of TT and ST, emphasize the role of the body in the processes of human understanding, and refuse the Cartesian dualism of body and mind: the basis for understanding lies already in the pre-reflective intentional connection between bodies; personal emotions and intentions are already present in any expressive behavior, which is therefore considered as meaningful from the very start (Thompson, 2007; Gallagher, 2001). Coherently with this perspective, Gallagher (2008b) notion of direct perception refuses the mind–mind gap (and therefore the mentalizing supposition) by claiming that other minds are directly perceivable in interaction: we can see grief or fear in the expression of another person without the need to infer or theorize. Perception is “smart”: when perceiving we already grasp the meaning of things in relation to us and our possibilities for action and response; this constitutes the basis of social understanding, which therefore mostly happens already at the pre-reflective, embodied level (Gallagher, 2008b). As Reddy (2008) reformulates it, emphasizing the role of emotional engagement, “we see feelingly.”

The idea that we need to develop a Theory of Mind (ToM) in order to understand others is challenged from a developmental perspective as well, as early processes of embodied intersubjective understanding have been shown to be already present during the first months of infants’ life (Trevarthen and Hubley, 1978; Trevarthen, 1979; Fivaz-Depeursinge and Corboz-Warnery, 1999; Reddy, 2008) and even in newborns (see Fivaz-Depeursinge and Philipp, 2014). This evidence stresses the role of emotional and pre-reflective engagement in social cognition (Reddy and Morris, 2004): the baby’s world is non-verbal. Developmental studies have clearly shown that infants learn to understand others, not via mindreading other persons’ independent qualities but through interactive engagement with them; for instance, the rhythmic attunement between the mother and the baby during breastfeeding is crucial for developing mind and communication (Kaye, 1982; Trevarthen and Aitken, 2001).

Another core assumption of the second personal stance lies in the refusal of the spectatorial supposition: we understand others in our everyday interactions with them, in the perception–action loops in which we are directly involved when interacting (Gallagher, 2008b). To believe that social cognition is based on an observational stance where we try to figure out the mental states of others as detached scientists does not do justice to our social reality. A second-person approach recognizes the intrinsic circularity of knowledge as a situated practice: “what we know of other minds must depend on our engagements with them, but these engagements must depend on what we know of them” (Reddy, 2008, pp. 31–32).

The process of social understanding cannot therefore be resolved by the sole effort of one person but it arises in the in-between of interaction, it is constituted by social interaction and shaped by emotional engagement (De Jaegher and Di Paolo, 2007; De Jaegher et al., 2010; Schilbach et al., 2013); moreover, as it takes two to tango, in order for an interaction to happen, the autonomy of the two interactors needs to be maintained (De Jaegher and Di Paolo, 2007).

With the concept of participatory sense-making (PSM) De Jaegher and Di Paolo (2007) emphasize the constitutive role of the interaction for social understanding, an aspect that has become a ground for criticizing and integrating Gallagher’s IT^11^ (De Jaegher and Froese, 2009; De Jaegher et al., 2010). In Reddy (2008) words: engagement in the interaction does not only provide information about minds but creates them. A similar stance is taken by Ugazio’s (1998, 2012/2013) constructionist approach, where she claims that conversational processes not only are the context in which individual identities develop but they are what constitutes them in the first place.

By stressing the importance of social interaction, it is, however, necessary to note that a second personal stance is not just a social constellation, the mere use of the “you,” rather being an attitude of openness that involves the recognition and acknowledgment of the other as a person; it requires that we directly address the other as someone that can respond and understand (Reddy, 2008). Drawing on Buber’s (1937) distinction between *I–Thou* relationship (second personal stance) and *I–It* relationship (third personal stance), Reddy (2008) notices that, even when interacting with someone, we may still regard him or her as an object, an instance of a category; with this stance, we do not take seriously the ongoing interaction and we actually remain in an observational, detached position.

As Fuchs (2012) also emphasizes, drawing on phenomenologists such as Husserl and Scheler, what distinguishes object perception and the perception of another person lies in a radically different attitude toward the object. Object perception is an enactive and dynamic process in which we immediately perceive things according to their affordances, for their predictability and the possibilities of action they offer to us^12^ (Gibson, 1979). However, when we are directed toward other persons, our perception is not just driven by Gibsonian affordances, we relate to others in a “personalistic attitude,” which means, we engage and resonate with them and we are responsive to their behavior, emotions, and intentions (Fuchs, 2012). Engagement, resonance, and responsiveness are therefore core defining aspects of a second-person perspective.

Importantly, this attitude toward others implies not only the recognition of similarity (as it is stressed by a first-person, simulationist approach), but also the acknowledgment of difference. In fact, in order to experience the other as a particular other to whom we are responsive, we need to recognize his or her difference from us, otherwise we would simply reduce their experience to our own (Reddy, 2008). This becomes clear from a phenomenological point of view (Zahavi, 2010, 2011) when considering the notion of empathy, which is regarded as constituting the core of pre-reflective social understanding. Empathy grounds an unmediated and non-inferential access to others’ experience; still it differs from the direct experiential access we have to our own mind (the focus is on the other, not on ourselves or on what it would be like to be in the other’s place). The notion of empathy, as it is understood in phenomenology, is truly second personal: we encounter others as embodied subjects, we are able to empathically grasp their experience, and still, the experience we make of them is different from their original experience (Zahavi, 2010)^13^. Indeed, as noted by Murray and Holmes (2014):

Husserl (1989: 170–180) characterizes intersubjectivity as Einfühlung—empathy—and Heidegger (1962: 153–163) writes of an ontological or prepredicative Mit-sein—‘being-with’ others—a hyphenated formulation that points to the prereflective experiential inseparability of these terms. “He [sic] who speaks enters into a system of relations which presuppose his presence and at the same time make him open and vulnerable” (Merleau-Ponty 1973: 17). (p. 13)

The different contributions to a second-person approach mainly focus on the pre-reflective, implicit level of experience, on the way we intuitively grasp the others’ state of mind by engaging with them in here and now encounters. Refusing TT and ST suppositions of universality, the second-person approach therefore maintains that social understanding happens primarily at the embodied, pre-reflective level of experience; as Fuchs and De Jaegher (2009) called it, it is based on a dynamic process of “mutual incorporation.”

In this regards, Dullstein (2012) critically pointed out that the second-person approach, by emphasizing the role of pre-reflective processes of understanding, may not yet provide an answer to the problem of how we actually understand others’ mental states. In her comment on Reddy’s and Gallagher’s theory, she questions the extent to which these theories explain the phenomenon of social understanding, as it is conceived in the cognitive science debate. As she stated for Reddy’s account:

The phenomena Reddy points to are well-known and hard to deny. Emotions do shape the way we experience each other. But the question is as to whether these phenomena help us to give new answers to the questions which the ToM debate is about: Do they allow us to acquire knowledge about the other’s feelings or beliefs? (p. 236)

Similarly, she criticizes Gallagher for confusing two different notions of understanding: namely, understanding others in terms of their mental states and understanding as basically engaging or interacting. Although engagement and interaction are important and constitutive for social understanding, they cannot be confused with it; contrary to what Gallagher (2008b) claimed, social cognition is not the same as social interaction (Dullstein, 2012).

These questions are particularly relevant for the issue at stake in this paper; in fact, although (as we shall later argue) the interaction and engagement with research participants is of core importance for a methodological reflection, the research enterprise in psychopathology aims at understanding patients’ meanings, beliefs, motives, and not just at empathically grasping them.

As Zahavi (2010) clearly outlines, drawing on Schutz’s insights:

Although on Schutz’s view it is permissible to say that certain aspects of the other’s consciousness, such as his joy, sorrow, pain, shame, pleading, love, rage and threats, are given to us directly and non-inferentially, he denies that it should follow from the fact that we can intuit these surface attitudes that we also have a direct access to the *why* of such feelings. But when we speak of understanding (the psychological life of) others, what we mean is precisely that we understand what others are up to, why they are doing what they are doing, and what that means to them. To put it differently, interpersonal understanding crucially involves an understanding of the actions of others, of their whys, meanings and motives. And in order to uncover these aspects, it is not sufficient simply to observe expressive movements and actions, we also have to rely on interpretation, we also have to draw on a highly structured context of meaning (Zahavi, 2010, p. 297).

By emphasizing the role of pre-reflective understanding, in which we can transparently grasp intentions and emotions of others, most exponents of the second-person approach (Gallagher, 2008b; Fuchs and De Jaegher, 2009; Fuchs, 2012) see this intersubjective endeavor as mostly unambiguous: “in our everyday engagements we do not constantly go around trying to solve puzzles” (Gallagher, 2008a, p. 169). However, they do not deny that behavior may actually become ambiguous in many situations and in these cases, since we cannot rely on primary embodied understanding, we need to start reflecting on the other’s mental states, motives, and intentions. This is the place where TT and ST still play a role in understanding: we may in fact need to assume a more detached stance toward others and try to infer or simulate their mental states in order to understand them (Gallagher, 2008a,b; Fuchs, 2012).

Ratcliffe (2006) argued against the need to go back to a first- or third-person perspective in order to explain higher level processes of understanding: “all instances of interpersonal understanding are interactive. A wholly detached, theoretical I-he/she/it stance is something that is never adopted towards persons. Even third person stances are interactive and should not be identified with the impersonal stance of scientific enquiry” (p. 42; see also Di Paolo and and De Jaegher, 2012). Taking seriously the constitutive role of the interaction process, which is one of the core assumptions of the second-person approach, Ratcliffe (2006) denies that even more reflective processes of understanding may be seen as a person attributing mental states or unidirectionally interpreting another person: “B is not just interpreted by A but is also constitutive of the process through which A interprets A, B and the relationship between them” (p. 40)^14^. Therefore, social cognition should be rather seen as a collaborative enterprise of mutual understanding about the persons involved, their beliefs, their experiences, and emotions (Dullstein, 2012). This process could be described, at the linguistic conversational level, as Gadamer’s (2004) hermeneutic circle: a mutual agreement, co-constructed in the interaction, on an object, which in this case is one of the persons involved. Similarly, at the implicit level, the same process may be understood, with Waldenfels (1979) as a mutual tuning of the two partners involved, as it happens, for example, in caregiver–infant proto-conversations (Dullstein, 2012).

As it is clear in Zahavi’s (2010) words, for understanding others we rely not only on pre-reflective processes of perception in the here and now encounter but also on interpretation and on “highly structured contexts of meaning” (p. 297). Social understanding and meaning-making do not happen in a vacuum: according to the British anthropologist and cyberneticist Bateson (1979), “without context, words and actions have no meaning at all” (p. 15). Therefore, depending on the context we are in, our behaviors, beliefs, and the meaning we attribute to our own and other people’s experiences and relationships may vary; and thus we may position ourselves and be positioned by others in different ways. Cronen et al. (1982) in the Coordinated Management of Meaning theory (CMM) showed how, in the context of the here and now situation, different levels of meaning intertwine and coordinate in a mutual interaction with others: starting from the episode and going up to the personal history, the history of the relationship and the cultural framework. All these aspects play a constitutive role in social understanding and come into play in every social encounter.

## METHODOLOGICAL IMPLICATIONS FOR A SECOND-PERSON PSYCHOPATHOLOGY

If we adopt a second-person perspective in understanding social cognition, what are the implications for the particular kind of interaction that is the focus of this paper, namely the relation between a researcher and a person presenting with a psychopathology? How may the insights coming from the social cognition debate enlighten the methodological process of research in psychopathology?

If we start from the last (and strongest) claim by Ratcliffe (2006), that any kind of interpersonal understanding is always constituted and influenced by the interaction in which it arises, we may first start to see that the research process is not as linear as it would seem. There is no epistemic subject (the researcher) gathering information about an epistemic object (the patient), but a dynamic process of sense-making in which both participants, as well as the interaction and its context (or setting), have a constitutive (although different) role. Interpersonal understanding conceived as a collaborative enterprise points to the active role of research participants in the constitution of knowledge and to the relational nature of the elicited data; even in experimental studies in psychology, participants’ behavior is always an answer to a question posed by the researcher (Rommetveit, 2003). Indeed, especially in psychopathology research, one needs to acknowledge that patients are not passive objects to be analyzed but, according to a second-person approach, they always contribute to the process of understanding. As Rommetveit (2003) puts it: “Coauthorship of psychological theory on the part of the human informant is an epistemologically unique and distinctive feature of the psychology of the second person as a communicative genre” (p. 212).

These considerations necessarily raise the issue of validity in psychopathology research: are our descriptions and theories actually about what we claim to be the object of our research (i.e., the patient’s experience)? If, as Rommetveit (2003) claims, psychology is a communicative genre, the data we elicit always contain information not only about the other, but also about ourselves. Moreover, drawing on Reddy (2008) account, we may push this argument even beyond the level of communication into the very pre-reflective process of perception:

Our perceptual experience of another person’s frown or smile or tears, therefore, must always include in it our proprioceptive experience of our own bodily state and, most importantly, our affective and motivational state. Conversely, our proprioceptive experience of our own acts and reactions and feelings always involves the perception of what relevant others are doing, saying or feeling. As the psychologist John Shotter put it, there is a constant intertwining and intermingling of the two (p. 30).

Although Reddy (2008) argues that within active emotional engagement this link between proprioceptive experience (of the self and of self-feelings-for-the other) and perceptive experience (of other-feelings-for-the-self) is much tighter than in uninvolved observation, she also reckons that this intertwinement still happens even in more disengaged stances. Methodologically, it is therefore necessary to acknowledge this link and, for the sake of validity, it is important to find ways to disentangle it.

In contrast to quantitative research methods that postulate the neutral observational position of the researcher, qualitative methods in psychology (and therefore in psychopathology research) acknowledge reflexivity: that is, the researcher, in gathering the data and producing the analysis, is always a constitutive and influencing part of the research process (Dallos and Vetere, 2005; Lyons and Coyle, 2007). This is a core methodological concern in qualitative research that is dealt with through different strategies: going from, as it is common for all qualitative methods, an explicit consideration of the researcher “speaking position” (i.e., the epistemological framework); up to finer techniques that allow a thoughtful inclusion of the researcher’s feelings, impressions and assumptions in the analysis process (as it is common to, e.g., Interpretative Phenomenological Analysis, Grounded Theory, or Narrative Analysis); and finally in actual cooperative (or co-authoring) research designs where the participant becomes actively involved in the process of validity check, for example, through respondent validation (Dallos and Vetere, 2005; Lyons and Coyle, 2007)^15^.

We consider the use of reflective practice, in its different forms and techniques, a very important methodological step for the research process. Reflecting on one’s own theoretical assumptions and research questions but also on one’s own personal motivation and personal history is a way of acknowledging the very intersubjective aspect of the research endeavor which does include the researcher as a constitutive part of it. Di Maggio et al. (2008) have interestingly maintained that autobiographical memory plays an important role in understanding others (especially with dissimilar others) and they therefore suggested that self-reflection may enhance the possibility and accurateness of social understanding^16^.

Another methodological implication of a second-person perspective, which again seems to be coherent with qualitative research methods, has to do with idiography. As already briefly mentioned in the previous section, Reddy (2008) highlighted the importance of acknowledging the other for his or her difference, avoiding reducing the other to a category or to his/her similarity to ourselves. From a second-person perspective, we see the other as a particular other:

A second person perspective pluralizes the other: there is no such ‘the other’ but different others depending of different degree and type of engagements. Engagement in the second person allows us to experience others within our emotional responses to them as particular others—an experiencing which is more than simply a recognition of their similarity to ourselves. (p. 27)

Similarly, idiography is concerned with the particular person: in contrast to nomothetic approaches, which are rather concerned with making claims at the population level and demonstrating general rules, idiographic approaches value the in-depth and detailed analysis of particular cases. There is no general “other,” that may be equated to an average or a category, but single persons and single encounters to be understood in their own right. It is not the case that idiography eschews generalization, only, the strategies for generalizing are different and the methodological focus is on validity rather than on reliability (Smith et al., 2009). A focus on in-depth analysis of single cases has also been stressed by phenomenology: “It is not so much the number of cases seen that matters in phenomenology but the extent of the inner exploration of the individual case, which needs to be carried to the furthest possible limit” (Parnas et al., 2013, p. 273). In this case, generalization is not based on statistical average but on the typicality of a case (a prototype). In fact, the most illuminating cases are often not the most common ones (statistically speaking) but rather the exceptional ones; in this sense, the generalization from these cases qualitatively provides an expansion of understanding on the studied phenomenon (Parnas et al., 2013).

As we have seen, the recognition and acknowledgment of the other person in his or her difference and uniqueness, the active role of the other person in the process of interaction and the constitutive influence of the very interaction process for social understanding are core claims of a second-person perspective that have important methodological implications. Though, a second-person approach not only makes us aware that the knowledge about the epistemic object comes from our relationship with it but also that this relationship is mainly played out at the embodied level of engagement and empathy, which constitutes the core of social understanding. As we have outlined, phenomenological approaches contributed to the social cognition debate by highlighting the role of direct, pre-reflective processes of understanding that take place in the actual encounter between embodied subjects.

Coherently, within the tradition of phenomenological psychiatry this emphasis on pre-reflective engagement and on the importance of empathy for understanding others emerged in techniques like “the feeling diagnosis,” where the clinician’s emotional reaction to the patient was considered a way to understand psychopathology (Reddy, 2008).

The relevance of the embodied here and now situation of the clinical interview has also been stressed by the more recent phenomenological approach of Parnas et al. (2013). They contrasted a phenomenological method of interviewing with standard structured assessments, underlying how the interaction between the interviewer and the patients should be structured as a mutually interactive reflection: a dialogical I–Thou situation where the interpersonal rapport is crucial for eliciting the patient’s experience in its full complexity and for understanding meaningful connections (Nordgaard et al., 2012). This stance is first of all based on a “phenomenological reduction”:

What a phenomenological interviewer attempts to do is to suspend the standard presuppositions of the shared, commonsense world, the unquestioned, commonsense background with its assumptions about time, space, causality, and self-identity, and about what does and does not exist as “real.” (Nordgaard et al., 2012, p. 360).

This first step allows the interviewer to be open toward the other and engage in a truly second personal and dialogical process of exploration, rather than monologically lead the interview according to predefined assumptions.

Notwithstanding the importance of this methodological shift, a second-person method cannot be limited to the here and now encounter between two embodied subjects. The intersubjective endeavor of the research process in fact does not end with interviewing but goes on through the whole process of analysis and a thorough methodological reflection on this process seems to be missing in contemporary phenomenological psychiatry.

As Dullstein (2012) noticed, the pre-reflective engagement, the acknowledgment of the other person in a truly second personal stance does not yet answer the question of how we understand the other person’s beliefs, intentions, and motives. Similarly, even if a phenomenological interview allows a much more detailed and coherent description of the other’s first-person experience, the question of how to understand these data still remains unanswered. In the here and now moment of encounter with the patient, the researcher, by bracketing his own assumptions, allows the opening of a space where the other’s experience can be freely elicited in its full complexity and, by taking an I–Thou stance in the interaction, he can have an implicit direct grasp of the patient’s experience. But how can we understand what we cannot immediately empathically grasp in the interaction? How can we make sense of the ambiguous or bizarre behaviors^17^ (which often lead the diagnosis of a psychopathology) that do not actually appear to be meaningful to most of us?

As mentioned above, in cognitive science, the problem of how to understand the other in ambiguous situations, when primary and pre-reflective intersubjective processes of understanding are not enough, was often solved through a shift from an implicit second-person stance to an explicit third or first personal, reflective stance. The same shift can be often witnessed in psychopathology research, when moving from the here and now interview situation to the actual process of data analysis.

For instance, the EASE interview (Parnas et al., 2005), created for exploring anomalous self-experience in schizophrenia, is based on a phenomenological second-person understanding of the interview process which allows a thorough exploration of the patient’s experience. Yet, the way this experience is accounted for in the analysis process seems to fall back into a third-person approach, since a checklist is used for evaluation. In fact, by using a checklist, the researcher reads the data (the elicited experience of patients) according to a “normative” theory, i.e., s/he looks for and selects the patient’s words that fit into his/her theoretical framework, which is defined *a priori*. By doing this, the researcher assumes an independent and neutral third personal stance. Although the EASE checklist is inspired by a phenomenological theory of schizophrenia, this does not ensure that the methodology is truly phenomenological or second personal.

We do not deny the usefulness of checklists and of third-person approaches in general. Sometimes they constitute a necessary step for the research process, which should ideally combine different methods or tools; we believe that methodological pluralism is the way to go. Nevertheless, when applying a third-person method, it is important to be aware of its implications and, as highlighted above, of the problems that come with it. Using a checklist to read through empirical data may indeed be a useful way to validate a theory; on the other hand, though, if the authority of the analysis process remains with the theory (as in the case of third-person methods) the risk is to fall into a tautological process, where a theory is built on a reading of empirical data according to the same theory. In order for a theory to develop further, we believe that a second-person stance is necessary (at least as a step in the research process) to re-allocate the authority of the analysis process to the other’s experience (see the end of this section for a further elaboration on this point).

Another example of this methodological issue is Davidson (2003) qualitative phenomenological analysis of interviews with persons with schizophrenia. As in the case of Parnas’ studies, Davidson’s interviewing technique is phenomenological, i.e., based on phenomenological reduction and on a dialogical second-person stance toward the other. The process of analysis though, seems to be rather first personal in the method that is applied for understanding the elicited narratives. This process is in fact mainly based on the concept of empathy, here conceived as an imaginative transposal into the other’s place:

In cultivating empathy for another person’s experiences, we have found it useful to build imaginative bridges between his or her experiences and our own. We do this—especially in cases in which the meaning of the experience is far from obvious—as one might do in certain acting classes, by recalling experiences in our own lives that have similarities to the experiences in question (Davidson, 2003, p. 123).

Although “stepping into the other’s place” is methodologically very important if we are to get as close as possible to participant’s original experience, the worry within a first personal method is still whether this is enough to grasp his/her “otherness,” i.e., the aspects of his or her experience that I would not grasp even if I were in his or her shoes, because I am a different person.

As highlighted in the above discussion on third- and first-person methodology, the Procrustean risk of walking down these routes is that we either try to fit the patient’s experience into our own theories (eventually leading to tautology) or reduce it by analogy to our own experience. Although we acknowledge the importance and value of both Parnas and Davidson’s work, with these two examples we wanted to show how, by grounding the validity of our understanding only on the here and now engagement with the patient (e.g., in the interview method) we may fail to account for his/her “otherness,” the aspects of his/her experience that we may not immediately grasp or empathically understand.

In order to overcome this methodological problem, Stanghellini and Rosfort (2013) proposed the notion of “second-order empathy,” as a valuable alternative that goes beyond both the phenomenological notion of primary non-conative empathy and the conative notion of empathy. Non-conative empathy is the most basic form of empathy: the pre-reflective resonance between my own and the other’s lived body that allows a direct, implicit understanding. Conative empathy is a more reflective and cognitive task that requires more than implicit attunement at the level of the lived body. Conative empathy is based on one’s personal past experiences and knowledge of commonly shared experiences (common sense), and it consists in an active reflective act of understanding by analogy: “I look inside myself for stored experiences to make them resonate with those of the other” (Stanghellini and Rosfort, 2013, p. 342). By contrast, second-order empathy does not rely on similarity or analogy with the other, rather being based on the recognition of the other’s autonomy: “In order to empathize with these persons, I need to acknowledge the existential difference, the particular autonomy, which separates me from the way of being in the world that characterizes each of them” (Stanghellini and Rosfort, 2013, p. 343).

Through the recognition of difference, the process of interpersonal understanding takes the form of a hermeneutic circle of negotiation of meaning between two autonomous subjectivities. Stanghellini (2010) therefore proposed hermeneutics as a framework for understanding psychopathology, which may be coherent with a second-person stance:

Second-person understanding, which requires an involvement (engagement) of the researcher (interviewer), but not of the kind that may obstruct the reliability of results, complements the first-person approach. It envisions understanding not as the effect of the empathy or the internal actualization of the other’s experience, but as an open cycle of questions and answers between interviewer and interviewee. Dialogue, seeking corroboration of the interviewer’s constructs and the interviewee’s self-understanding, is the major method of inquiry for structural psychopathology. (pp. 323–324)

As Blankenburg (1980) stressed, although from a phenomenological stance the researcher tries to bracket his own assumptions in order to get as close as possible to the other’s experience (trying to grasp it in its own autonomy), it is inevitable that one’s own subjectivity enters in the process of interpretation.

An integration of phenomenology and hermeneutics has already been recognized as pointing in the direction of a second-person methodology, although the combination of the two has been so far rather unsatisfactory; in fact, hermeneutics has been only considered mainly for its role in interviewing techniques (Stanghellini, 2007, 2010) or in psychotherapeutic praxis (Fuchs, 2010). Instead, we argue that hermeneutics (together with phenomenology) should be taken seriously for a methodological grounding of the process of understanding at play in psychopathology research.

Integrating phenomenology and hermeneutics, Smith et al. (2009; see also Smith, 2004) developed a method of analysis, Interpretative Phenomenological Analysis (or IPA), that we propose here as an example of a second-person methodology that may be a valuable tool for psychopathology research. Without going into the technical details of IPA, we consider IPA as a valid and non-reductive attempt to grasp the other’s experience: namely, a dynamic understanding that goes from the *within* (the patient’s experience) to the *between* (the researcher and the patient) and back.

The dual process of understanding in IPA unfolds through a *double hermeneutics*, i.e., a circular movement like a dance, where, on one hand, we (try to) bracket our own prejudices and we empathically engage with the other, taking on an insider stance led by a *hermeneutics of empathy* (Smith et al., 2009); on the other hand, we use our own impressions, feelings, theoretical assumptions, and even critical stance for interpretation (*hermeneutics of questioning*). This accords well with what Reddy (2008) has argued, namely that a second-person methodology needs to be balanced between engagement and disengagement, being involved and at a distance, stepping into and out of the frame to explore it better.

In this dual process, we temporarily try to suspend (or better, bracket away, in the sense that they are acknowledged and tracked down, not ignored) our own personal lens to become more sensitive to the experiences of the other during both interviewing and analysis. When reading the transcripts, we note different kinds (descriptive, linguistic, interpretative, and self-reflexive) of comments at both margins of the text and we make use of a research journal to track and bracket our thoughts that may be later integrated in the interpretation. To put it in Smith et al.’s (2009) terms:

By focusing on attending closely to your participant’s words, you are more likely to park or bracket your own pre-existing concerns, hunches, and theoretical hobby horses. It is not that you should not be curious and questioning; it is that your questioning at this phase of the project should all be generated by attentive listening to what your participant has to say. (p. 64)

The second step of the analysis process is rather interpretative: we do make sense of the other’s experience from our personal stance and theoretical framework. However, if we are to avoid a third-person theorizing stance, interpretation cannot be based on a hermeneutics of *suspicion*^18^ (Smith et al., 2009), where we understand the other’s experience according to a theoretical perspective from the outside (an outsider expert stance, as for instance in psychoanalysis): the authority that should give validity to our claims is the experience of the other (Smith et al., 2009).

Interpretation is therefore here a reading from *within* the participant’s experience^19^, yet, it emerges out of a continuous process of interaction *between* the researcher and the participant in the situated context, as meaning making does not happen in a relational void.

Coherently with a second-person perspective, Brown et al. (2011) contend that IPA provides a valuable alternative to various research methodologies that fail to account for the lived totality of individual experience, which is often either fragmentized and broken into separate components (e.g., cognition, emotion, memory, personality) or reduced to other analytic frames at broader social levels (e.g., discourse analysis).

However, this approach also has its limitations. First of all, it often fails to grasp the embodied level of meaning-making which lies at the core of any phenomenological encounter: what Brown and colleagues have called “the methodological problem of body in psychology” (Brown et al., 2011, p. 496; Cromby, 2012). To borrow Murray and Holmes’ (2014) words:

And yet our impression of the IPA literature was that the body itself is often absent, or simply presumed to exist behind straightforward descriptions (or spoken testimony) from research participants, as if these descriptions straightforwardly conveyed what is called the lived-experience of the subject, his/her body, and his/her intersubjective relations with others. (p. 6)

Although a detailed methodological discussion of IPA is outside of the scope of this paper, this criticism is worth mentioning here as it touches upon one of the core aspects of a second-person approach: the primary embodied and pre-reflective processes that are always at play in social understanding.

Murray and Holmes (2014) recall Merleau-Ponty’s (1973) original concepts of the embodied *parole parlante* (speaking speech) as opposed to *parole parlée* (spoken speech). Whereas the focus on “spoken speech” may seem to embrace the Cartesian reduction of the body to a lifeless object/matter (i.e., Husserl’s *Körper*), Merleau-Ponty’s phenomenology aims at understanding the embodied language rather than the abstract and decontextualized text: body and language are intertwined and inseparable. The participant’s text is always embedded in the lived experience, its original context(s), and in the context of the intersubjective interview itself.

In most qualitative methods for analyzing interviews (IPA included) the “speaking speech” is often accounted for through the use of meticulous and accurate transcription procedures, which typically include taking notes on the participant’s most evident para- and non-verbal behaviors (e.g., pauses, smiles, and crying) during the interview by inserting them into square brackets and, where relevant, commenting shortly on the episode. This practice has been criticized for failing to grasp the full embodied and intersubjective experience as situated:

It remains a (formalized, methodologically constrained) way of translating embodied experience into language: as such, it is just as likely to omit something of its ineffable quality as any other such attempt (…) it leaves the gulf between language and embodied experience intact whilst nevertheless giving the superficial appearance of bridging it. In this instance, then, it can appear as though embodiment has been addressed through the technical accumulation and management of detail (Brown et al., 2011, p. 499).

Brown et al. (2011) suggest that rather than seeking the solution in transcription techniques, the methodological issue of the body needs to be addressed differently. In this regard, in our opinion it is worth noticing a particular technique often implemented in qualitative methods: the recollection of interview (otherwise also referred to as diary of interview or research journal). The recollection of interview is the first stage of IPA, where the researcher writes down all his or her immediate impressions, feelings, thoughts that arose in the embodied encounter of the interview situation. If then integrated in the analysis process^20^, the recollection of interview may be seen as a better way to account for the “speaking speech” as well for the intersubjective context of the participant’s words.

Interesting alternative ways of analyzing lived experience in its full complexity (and not just as straightforward description of experience) may be found in attempts to look not only at the content level of what is narrated but also at the way contents are talked about in the situated interaction. For instance, Lysaker et al. (2002, 2003, 2005) put a particular focus on aspects like the coherence and quality of narratives for understanding patients’ experience. Similarly, Seikkula et al. (2011) focused on the dialogical quality of therapeutic conversations for investigating the experience of change. A further remarkable example of this research strand is put forward by Ugazio et al. (2009, in press): in their analyses of therapeutic conversations they not only looked at the “narrated” meanings but also at the “narrating” and “interactive” levels, which refer to the more implicit, embodied and interactive dimensions.

Other methods have tried to include the embodied aspect of communication in the analysis process, as for instance the PRISMA method (Pieper and Clénin, 2010): a video-supported analysis method that uses the sensations, emotions, and thoughts of the researchers as tools for understanding.

Although some steps have been already made in this direction, the “methodological problem of the body” in understanding the other’s experience seems to be still an open issue that needs to be accounted for, especially in regards to methodologies coherent with a second-person approach.

## CONCLUSION

In this paper, we have critically reviewed the main theories at the heart of the social cognition debate: looking into the core principles of the third-, first-, and second-person approaches, we have highlighted the implications and limitations of each theoretical stance. Moreover, we have outlined how the second-person perspective addresses and tries to solve different problems related to third- and first-person theories of social cognition. Following the different contributions to this debate we have also stressed how, even within a second-person proposal, some issues still remain controversial; indeed, the second-person approach does not yet provide a definitive answer to the dilemma of social understanding, but in our opinion it represents the most convincing account of social cognition put forward so far within this field.

We followed Reddy (2008) in maintaining that the problem of understanding others constitutes the core methodological issue of psychology research and that therefore the theoretical frameworks accounting for this problem should inform the very process of research in its methodological concerns: we do try to understand others when doing research in psychology. Thus, we decided to focus this paper on psychopathology research, making it a paradigmatic case of this methodological issue.

Accordingly, linking social cognition theories with research methods based on similar assumptions, we underscored how the shortcomings and implications of each theoretical stance could be also viewed as methodological problems in psychopathology research. Once the epistemological and theoretical frame are recognized and explicated, third- and first-person methods can be criticized according to the same arguments that deconstructed these perspectives in the social cognition debate: i.e., mainly, the assumption that, for understanding others, the researcher starts from an observational stance, which is detached and independent from the object of study; that this stance is in the first place observational and that therefore processes of understanding occur within the observer (denying the primary and founding role of the interaction in meaning making); that the primary processes of understanding are already based on theorization and inference, leaving out the immediate embodied level of engagement and direct perception (instead of emerging from the dynamic intersection between both levels); and (for a first-person approach) that social understanding is based on my own individual experience, in analogy with the other’s, but disconnected.

By discussing and challenging these assumptions, we outlined what we consider to be the core principles of the second-person approach, drawing on the different contributions that constitute it: i.e., mainly, the recognition of embodied and more direct processes of social understanding as primary (and therefore the importance of non-conative empathy and engagement for understanding); the assumption that our everyday stance toward others is not observational but interactive; the importance of the social interaction process as constitutive for social understanding; the fundamental personalistic attitude we assume toward others as soon as we recognize them in their difference and we acknowledge them as responsive others (an attitude that is here seen as a pre-condition for social understanding). Besides, we support the claim that the intersubjective matrix of social understanding does not simply draw on the implicit immediate level of interaction but also on higher and reflective intertwined levels of meaning, that are therefore seen as unfolding in the form of a hermeneutic circle.

Finally, we looked into how second personal theories of social understanding can inform the epistemology and methodology of psychopathology research, by reviewing research principles, techniques, and methods that are coherent with this perspective. We do believe that a second-person perspective is the most convincing methodological framework so far put forward for psychopathology research as it best accounts for the validity of our claims about the other.

The aim of this paper though, is not to defend one particular research approach against another, but rather to point out the different theoretical and epistemological assumptions supporting each methodological stance; therefore we critically discussed the limits and implications of different research methods. Although an integration of different techniques is needed and useful in research, and even first- and third-person methods should not be totally rejected, the problem of methodological pluralism centers on how to integrate methods in a complementary and meaningful way, so that we can preserve the validity of our final claims.

First of all, we believe that, in order to integrate different methods properly, a stronger critical awareness of their epistemological underpinnings, and their different targets and limits is needed. The reflections and discussions outlined in this paper are aimed at drawing the attention to this important issue, to enhance this awareness, or at least to offer some inputs for further debate. Secondly, if we are to avoid a view of methodological pluralism as a clash of (sometimes even contradictory) methods, research in psychopathology should be conceived within a broader theoretical framework addressing the problem of how we get to know others in the first place.

For instance, Reddy, 2008 maintained for a second-person approach:

Disengagement is not only inevitable, it provides a valuable dimension to knowledge that is born within engagement. Buber, comparing the intense intimacy of the I-Thou way of knowing with the I-it way, pointed out (albeit poetically) the inevitability of the latter: genuine engagement for him was a time-limited phenomenon. (…) But this is not detachment; it is disengagement born within and alternating with, engagement. What psychological science need is a balance—engagement first and disengagement second—between the two. (pp. 34–35)

Similarly, according to a second personal framework, we can argue for the need to integrate quantitative methods (third-person stance)^21^ with qualitative ones (first- or second-person stance) in psychopathology research; but, for this integration, the validity of the results should rely on the latter, rather than on an illusory objectivity of the first; as Reddy wrote for a second-person perspective, engagement comes first.

In this regard, we believe that a second-person framework should always inform psychopathology research, as in the end we can only know others intersubjectively, from the more embodied levels of participatory sense making in the here and now encounter, to the hermeneutic circles of interpretation where different contextual levels of meaning come into play.

### Conflict of Interest Statement

The authors declare that the research was conducted in the absence of any commercial or financial relationships that could be construed as a potential conflict of interest.

## Footnotes

1 *Mind* is here not conceived in the narrow sense of a computational mind or brain but rather, in a broader sense, as the subject matter of cognitive science. Similarly, we refer here to social understanding as an epistemological “problem,” not in the narrow sense of a gap between persons that needs to be filled; irrespective of the theoretical framework we embrace, understanding others remains a complex phenomenon that needs to be better understood and therefore, in this broader sense, it does remain a dilemma.

2 *Different* is here understood not in an ontological or moral sense but simply in a phenomenological sense: we perceive and experience others as different, especially in the case of what we categorize as psychopathology. Making sense, defining, or deconstructing this difference is the very starting point of psychopathology research.

3 The idea that psychopathology can be assimilated to a “science of meaning” was originally formulated by Guidano (1991, p. 59) and is at the core of Ugazio’s (1998, 2012/2013) socio-constructionist model. This model lays the foundations for a systemic theory of personality development that explains the transition from “normalcy” to psychopathology by the reciprocal positioning that the individual and the persons meaningful to him/her take within the critical meaning.

4 According to these more critical stances, psychopathology is a stigmatizing label used to categorize, reify and medicalize the diversity and alterity of the other with the main outcome of pathologizing, alienating, and segregating them (see, for example—among the many—the criticisms put forward by classic authors such as Goffman, Laing, Foucault, Szasz, and more recently, Newnes, Parker, Timimi).

5 Despite the fact that clinical and research encounters are two different joint hermeneutic endeavors and thus have many different characteristics and features, they also share many epistemological and methodological aspects and dilemmas (e.g., power and knowledge gap, reciprocal positioning), especially if we consider more recent collaborative–participative research designs employing in-depth qualitative and creative interview methods.

6 In the TT approach, minds and mental states are considered in a “software–hardware” relation to the body (Thompson, 2007); cognitive processes therefore happen within Cartesian minds, conceived as radically separated from the body (body–mind dualism) and from other minds (Western individualism).

7 According to TT, these mentalizing processes constitute our primary and pervasive way of understanding others (supposition of universality).

8 Here the concept of consciousness is not meant in a psychodynamic sense, i.e., as opposed to the unconscious, but in a phenomenological sense: see quote from Parnas et al. (2013) below for a more detailed definition.

9 And also interpersonal functions or purposes, according to a more systemic–relational perspective.

10 We are aware that the view presented here is just one possible interpretation of Jaspers’ General Psychopathology. Indeed, our aim here is not to engage in a critical discussion of Jasper’s work or to identify this particular reading with phenomenological psychopathology in general, but to present an example of what a first-person methodology in psychopathology research may mean.

11 As Schilbach et al., 2013 also noted, in fact, Gallagher’s initial notion of direct perception may still fall into an observer epistemology: knowing other minds means perceiving them.

12 Indeed Gallagher (2008b) maintained that perception is “smart” even when directed to material things: “I do not see red mass, shape, and color, and then try to piece all of that together to make it up to my car. I simply and directly see my car. (…) I see the car not just as some object among others, but as an object that I can use—that I can climb into and drive.” (pp. 356–357)

13 Upholding the recognition of an irreducible otherness, the concept of empathy cannot be clashed with a first person, simulationist approach. For a more detailed discussion on the topic, see Jardine, J. (forthcoming).

14 Ratcliffe’s claim touches upon the core underpinning of social constructionism—although phenomenology differs from social constructionism in its ontological and epistemological claims—that is, the role of conversational processes as constitutive for social understanding; these claims are therefore also tightly linked to ideas of circular causality as put forward by cybernetics and systemic thinking.

15 Importantly, as Davidson (2003) noticed, in psychopathology research, to include the patient as a partner in the research enterprise does not mean to consider him/her as a fellow scientist: “the role of the participant in our research is not to be a fellow psychologist but to be precisely what she or he is: (e.g.) a person experiencing life with schizophrenia.” Cooperation with research participants is indeed based on the recognition of differences and of different roles.

16 We do not agree with the simulationist approach proposed by Di Maggio et al. (2008) and with the emphasis they put on processes of mentalizing and mindreading. Yet, we believe that some of the insights proposed in their paper may be interesting even if looked at from a different theoretical framework and if applied to methodological issues.

17 By this expression we refer to those experiences that in most cultures are perceived and/or defined as extraneous to common sense understanding, e.g., psychotic experiences, hallucinations and delirium, although this is at the core of an animated debate.

18 Smith et al. (2009) draw on Ricoeur’s (1970) distinction between two opposed interpretative positions: the hermeneutics of empathy and the hermeneutics of suspicion. Whereas the first attempts to reconstruct the original experience in its own terms, the latter is based on theoretical perspectives form the outside for understanding the phenomenon. Smith et al. (2009) therefore argue for a center-ground position that combines the two.

19 What Smith et al. (2009) call “a reading from within” was already mentioned by Blankenburg (1980) as “immanent interpretation.” (p. 67)

20 Although an integration of the recollection of interview in the analysis process is coherent with the IPA guidelines, this technique is not always implemented. In fact, because the IPA guidelines are quite flexible, many methodological decisions are left to the researcher’s judgment.

21 Seen here as relying on a stance of “disengagement” rather than “detachment,” according to Reddy (2008) distinction.
